# Mortality trends in chronic obstructive pulmonary disease in 27 countries within Europe from 2011 to 2021

**DOI:** 10.1136/bmjresp-2025-003175

**Published:** 2026-01-05

**Authors:** Ayushman Gupta, Francesca Gonnelli, Tricia M McKeever, Rachael L Murray, Richard Hubbard, Charlotte E Bolton

**Affiliations:** 1 NIHR Nottingham BRC Respiratory Theme, Translational Medical Sciences, School of Medicine, University of Nottingham, Nottingham, UK; 2 Respiratory Unit, Department of Biomedical Sciences and Public Health, Polytechnic University of Marche, Ancona, Italy; 3 Lifespan and Population Health, School of Medicine, University of Nottingham, Nottingham, UK

**Keywords:** COPD epidemiology

## Abstract

**Background:**

Chronic obstructive pulmonary disease (COPD) incurs significant mortality worldwide. Less is known about the burden in the last decade across Europe. We report trends and variations in mortality for patients with COPD across 27 European countries from 2011 to 2021.

**Methods:**

COPD mortality was extracted from the EUROSTAT database, using the International Classification of Diseases 10 codes J43 and J44 for each country. Age-standardised and sex-standardised mortality rates (SMR) were calculated and joinpoint regression identified average annual percentage change (AAPC) in deaths from 2011 to 2021. Global Burden of Disease tobacco prevalence data were used to try and best contextualise the mortality.

**Results:**

The overall SMR in Europe for this period was 32.1 (95% CI 32.0 to 32.1) per 100 000 person-years, with substantial geographical heterogeneity. There was a fivefold difference in mortality rates between the countries with the greatest versus the least deaths. Although there was an apparent 3% (95% CI −4.4% to −1.6%) decrease in average annual deaths from 2011 to 2021 across Europe, there was no significant change in deaths from 2011 to 2018, prior to the UK leaving the dataset (a noticeably high outlier in SMR) and the COVID-19 pandemic. A 2% reduction (95% CI −2.6% to −1.2%) in annual mortality rate was noted in males from 2011 to 2018, while females increased (AAPC 1.3% (95% CI 0.1% to 2.6)%) in the same time frame.

**Conclusion:**

The plateau in COPD-related deaths across Europe from 2011 to 2018 demands focus. Geographical variation in mortality suggests under-reporting in some countries, which may underestimate the true burden.

**WHAT IS ALREADY KNOWN ON THIS TOPIC**

- Previous mortality estimates suggested declining trends in chronic obstructive pulmonary disease (COPD)-related annual mortality rates across Europe from 1990s to 2010. However, more recent statistics from directly reported national death registration data are limited.

**WHAT THIS STUDY ADDS**

- This study provides reliable, up to date COPD-related mortality statistics using a centralised European statistics institution dataset, EUROSTAT. The analyses highlight plateauing of mortality rates across European countries between 2011 and 2018 as well as considerable intercountry heterogeneity in deaths, which warrant further attention.

**HOW THIS STUDY MIGHT AFFECT RESEARCH, PRACTICE OR POLICY**

- The results from the study support the need for standardised inter-European public health initiatives to curb the burden of COPD.

## Introduction

 Chronic obstructive pulmonary disease (COPD) is the third leading cause of death worldwide.^1^ Although the health burden of COPD is disproportionately greater in low to middle-income countries,^2^ there is marked morbidity and mortality even in high-income countries. This incurs a large health, welfare and economic toll.^3 4^ Across Europe, previous studies have reported a decreasing trend in mortality rates from 1990s to 2010,^5 6^ likely reflecting the public health initiatives targeting smoking cessation and advancements in therapeutics. More recent COPD-related mortality trajectories have been published. However, many of these are based on statistical modelling frameworks that produce aggregated estimates.^7^,^9^ Country-specific statistics using directly reported national death registration data are limited across Europe in recent times.

We describe trends and geographical variations in mortality rates for patients with COPD in Europe by calendar years 2011–2021, using the European Statistics Institution (EUROSTAT) dataset. The main objectives are to present pooled mortality rate estimates and the annual trend in death rates across the European countries as well as stratified by age, gender and country. To add context, current smoking prevalence across each of the European countries was also assessed using the best available dataset.

This will provide reliable, up to date statistics derived from a centralised European registry to inform ongoing targeted public health decision-making and policy development to improve overall disease burden.

## Methods

This manuscript is written in accordance with the REporting of studies Conducted using Observational Routinely-collected health Data (RECORD) guidelines.

### Patient and public involvement

The importance of mortality trends linked to COPD has repeatedly been raised in prior discussions with patient groups and at public meetings. It was not appropriate to involve patients or the public in the design or conduct of the research. However, there is a plan for patient and public involvement to facilitate dissemination back to patient groups in a manner, deemed appropriate.

### Dataset

#### EUROSTAT dataset—mortality

EUROSTAT is a publicly available European statistical database containing principal cause of mortality data (1a, primary cause of death) derived from death certificates (https://www.ec.europa.eu/). It publishes data for European Union (EU) member states, European fair trade association countries and some candidate countries. The coding is carried out centrally in the EUROSTAT statistics office and checks of the quality of the data are implemented to optimise the validity of the data.^10^ For this temporal analysis, mortality data from 27 out of 34 available European countries (Austria, Belgium, Bulgaria, Czech Republic, Croatia, Denmark, Estonia, Finland, France, Germany, Greece, Hungary, Ireland, Italy, Lithuania, Latvia, Luxembourg, Netherlands, Norway, Poland, Portugal, Slovenia, Slovakia, Spain, Sweden, Switzerland and UK) were extracted from the dataset from 2011 to 2021. Data from seven countries (Serbia, Turkey, Iceland, Romania, Malta, Cyprus and Liechtenstein) were missing for half of the analysis time frame or more, and so these countries were excluded from the analysis. Deaths related to COPD and emphysema for people over the age of 40 years were extracted using International Classification of Diseases 10th edition codes J43 and J44 as well as additional disease demographic data (overall European population, stratified by sex, age and country). There were missing data for Bulgaria from 2011 to 2012, Greece from 2011 to 2013 and UK from 2019 to 2021.

#### Contextualising the mortality data

In an attempt to contextualise the mortality to smoking habits, data on prevalence of current smokers across the 27 European countries were extracted from 2011 to 2019 from the global burden of disease (GBD) (the data are available from GBD 2019 public datasets https://ghdx.healthdata.org/record/ihme-data/gbd-2019-smoking-tobacco-use-prevalence-1990-2019). The prevalence of smoking tobacco use was an age standardised point prevalence of current smoking, not a lifetime exposure or capturing ex-smokers.^11^

### Statistical analysis

The statistical analysis was conducted using STATA V.18 (StataCorp, Texas). The Surveillance Epidemiology and End Results statistical software (Joinpoint Regression Program, V.5.2) enabled segmented regression analysis to quantify trends in mortality. The average annual percentage change (AAPC) in mortality rate was a summary measure of the overall trend for a prespecified fixed time interval, while the annual percentage change (APC) reflected trends of specific segments and enabled estimation of trend changes within the entire analysis time frame. The methodology for the regression analysis is previously reported by Gonnelli *et al*.^12^ For the mortality data, the annual crude mortality rates were calculated across Europe as well as each country separately. The age and sex-standardised mortality rates (SMR) were estimated using direct standardisation, details of which are previously reported,^12^ with the European population in 2018 as the reference population (online supplemental table S1). The SMR was also stratified by sex and age (5-year age bands between 40 and 85 years). The total resident population for each year and for each country was used as the denominators. For the segmented regression model, the SMR was the dependent variable and year of death was the independent variable. The AAPC provided estimates of mortality trends over time for each country and Europe overall as well as the sex and age strata.

Data on current smoking prevalence were presented by country and year as well as averages over all of European countries in this study from 2011 to 2019. Percentage change in current smoking prevalence from the initial year to the final year of the dataset allowed estimation of trends over time.

### Sensitivity analysis

To account for the missing data, a sensitivity analysis with measures of SMR and trends over the years was carried out with the entire UK data being excluded from the analysis. A subgroup regression analysis on average annual mortality rates was also conducted for 12 countries with a population of more than 10 million people (Belgium, Germany, United Kingdom, France, Italy, Greece, Spain, Poland, Netherlands, Sweden, Czech Republic and Portugal) to further confirm the validity of the dataset.

## Results

### Mortality rates

Overall, there were 1 589 916 recorded COPD-related deaths (929 672 deaths in males and 660 244 in females) (online supplemental table S2). Across all countries from 2011 to 2021, the crude mortality rate was 31.0 (95% CI 30.9 to 31.0) per 100 000 person-years (online supplemental table S3). The age-SMR and sex-SMR for each European country followed similar patterns to the crude rates, and the intercountry variations were not changed by the standardisation process (SMR 32.1 (95% CI 32.0 to 32.1) per 100 000 person-years) (table 1a,b). Mortality rate was greater in males than females and also increased with older age strata across all of Europe (table 2a,b).

**Table 1:** Age-standardised and sex-standardised COPD-related mortality rates stratified by 27 countries in Europe and by years (a: 2011–2016 and b: 2017–2021)

(a)												
Country	2011		2012		2013		2014		2015		2016	
	Standardised rate	95% CI	Standardised rate	95% CI	Standardised rate	95% CI	Standardised rate	95% CI	Standardised rate	95% CI	Standardised rate	95% CI
Austria	32.8	31.5 to 34.2	33.4	32 to 34.7	34.7	33.4 to 36.1	32.3	31 to 33.6	35.8	34.4 to 37.1	33.7	32.4 to 35
Belgium	41.8	40.5 to 43.1	44.3	43 to 45.6	43.0	41.7 to 44.3	38.3	37.1 to 39.5	40.6	39.4 to 41.8	38.3	37.1 to 39.5
Bulgaria					20.5	19.4 to 21.7	19.9	18.8 to 21.1	22.1	20.9 to 23.3	17.7	16.7 to 18.8
Czech Republic	30.5	29.3 to 31.8	29.0	27.8 to 30.3	40.2	38.8 to 41.7	33.8	32.5 to 35.1	39.6	38.2 to 41	34.4	33.2 to 35.7
Croatia	46.7	44.2 to 49.2	30.8	28.7 to 32.1	31.5	29.7 to 33.8	49.5	47 to 52	53.1	50.6 to 55.6	47.9	45.6 to 50.3
Denmark	74.8	72.2 to 77.4	75.2	72.7 to 77.8	75.9	73.3 to 78.4	71.8	69.3 to 74.2	71.6	69.1 to 74	71.1	68.7 to 73.5
Estonia	19.1	16.1 to 22.1	20.3	17.3 to 23.3	19.4	16.5 to 22.2	21.5	18.5 to 24.5	19.8	16.9 to 22.6	19.5	16.8 to 22.3
Finland	25.5	24 to 27	24.9	23.5 to 26.4	23.7	22.3 to 25.1	23.1	21.7 to 24.4	23.8	22.4 to 25.1	22.9	21.6 to 24.2
France	13.5	13.2 to 13.8	14.1	13.8 to 14.4	14.4	14.1 to 14.7	13.2	12.9 to 13.5	14.7	14.4 to 15	14.3	14 to 14.6
Germany	36.2	35.8 to 36.7	36.4	35.9 to 36.8	38.6	38.1 to 39	35.4	35 to 35.8	39.6	39.2 to 40.1	37.1	36.7 to 37.6
Greece							24.9	24 to 25.9	29.9	28.9 to 30.9	24.9	24 to 25.8
Hungary	38.4	35.1 to 41.4	60.2	58.3 to 62.1	62.9	60.9 to 64.9	57.0	55.3 to 58.7	67.5	65.6 to 69.3	57.8	56.1 to 59.5
Ireland	57.1	54.1 to 60.2	60.3	57.2 to 63.4	60.4	57.4 to 63.5	55.2	52.4 to 58.1	57.8	54.9 to 60.7	55.8	53 to 58.6
Italy	30.2	29.8 to 30.7	30.9	30.5 to 31.3	28.3	27.8 to 28.7	27.1	26.7 to 27.5	28.9	28.5 to 29.3	30.0	29.6 to 30.4
Lithuania	31.9	29.4 to 34.4	31.0	28.6 to 33.5	32.0	29.5 to 34.4	26.0	23.8 to 28.2	28.1	25.9 to 30.4	23.8	21.8 to 25.8
Latvia	16.0	13.9 to 18.2	16.2	14 to 18.3	16.4	14.3 to 18.5	16.0	13.9 to 18.1	14.7	12.7 to 16.7	18.1	16 to 20.3
Luxembourg	39.4	32.5 to 46.3	39.3	32.7 to 45.9	34.8	28.7 to 40.9	30.1	24.6 to 35.6	37.2	31.2 to 43.2	32.4	26.9 to 38
Netherlands	51.7	50.5 to 53	54.5	53.2 to 55.8	50.2	49 to 51.4	42.8	41.7 to 43.9	50.0	48.8 to 51.1	45.8	44.7 to 47
Norway	51.4	49.1 to 53.7	54.6	52.3 to 57	52.7	50.4 to 55	50.0	47.7 to 52.2	52.9	50.6 to 55.2	53.0	50.8 to 55.3
Poland	29.0	28.3 to 29.7	26.7	26.1 to 27.4	27.7	27 to 28.4	22.6	22 to 23.2	24.6	23.9 to 25.2	21.6	21.1–22.2
Portugal	22.2	21.2 to 23.1	23.6	22.6 to 24.6	21.2	20.3 to 22.1	21.5	20.6 to 22.4	23.2	22.2 to 24.1	22.7	21.8–23.6
Slovenia	29.3	26.4 to 32.2	27.2	24.4 to 29.9	22.9	20.5 to 25.3	22.4	20.1 to 24.8	22.8	20.5 to 25.1	21.6	19.4–23.8
Slovakia	29.2	27.2 to 31.2	32.0	29.9 to 34.1	22.1	20.4 to 23.8	23.5	21.8 to 25.2	25.7	23.9 to 27.5	24.3	22.6–26.1
Spain	33.8	33.3 to 34.4	34.9	34.3 to 35.4	30.7	30.2 to 31.2	30.1	29.6 to 30.6	31.8	31.3 to 32.3	27.6	27.1–28.1
Sweden	31.4	30.2 to 32.6	32.9	31.7 to 34.2	31.5	30.4 to 32.7	30.7	29.5 to 31.8	31.4	30.3 to 32.6	32.5	31.4 to 33.7
Switzerland	26.8	25.6 to 28.1	27.4	26.1 to 28.6	28.4	27.2 to 29.7	26.2	25 to 27.4	27.7	26.4 to 28.9	25.6	24.4 to 26.7
UK	62.2	61.5 to 63	57.0	56.3 to 57.6	57.6	57 to 58.2	55.3	54.7 to 55.9	58.7	58.1 to 59.4	57.3	56.7 to 57.9
All Europe	34.7	34.5 to 34.9	35.0	34.8 to 35.1	34.9	34.7 to 35	32.3	32.1 to 32.4	35.1	35 to 35.3	33.3	33.1 to 33.5

(b)												
Country	2017		2018		2019		2020		2021		2011–2021	
	Standardised rate	95% CI	Standardised rate	95% CI	Standardised rate	95% CI	Standardised rate	95% CI	Standardised rate	95% CI	Standardised rate	95% CI
Austria	36.8	35.5 to 38.2	35.9	34.6 to 37.2	36.0	34.7 to 37.3	34.7	33.5 to 36	31.0	29.8 to 32.2	34.2	33.8 to 34.6
Belgium	38.5	37.3 to 39.7	38.5	37.3 to 39.7	37.8	36.6 to 38.9	33.6	32.6 to 34.7	31.6	30.6 to 32.7	38.5	38.2 to 38.9
Bulgaria	16.9	15.8 to 17.9	17.3	16.3 to 18.3	19.9	18.8 to 21	21.6	20.5 to 22.8	19.3	18.2 to 20.4	16.7	16.4 to 17
Czech Republic	36.8	35.5 to 38.1	35.4	34.1 to 36.6	34.2	32.9 to 35.4	31.7	30.5 to 32.9	30.4	29.3 to 31.5	34.2	33.8 to 34.6
Croatia	51.5	49.1 to 53.9	47.9	45.6 to 50.2	48.9	46.6 to 51.2	41.9	39.9 to 44	39.9	37.8 to 41.9	50.7	50 to 51.5
Denmark	73.6	71.2 to 76.1	74.1	71.7 to 76.5	67.7	65.4 to 70	62.6	60.4 to 64.7	65.8	63.7 to 68	70.8	70.1 to 71.5
Estonia	17.6	15 to 20.1	18.6	15.9 to 21.2	15.3	12.9 to 17.7	14.5	12.2 to 16.7	15.7	13.3 to 18	18.3	17.5 to 19.1
Finland	23.0	21.7 to 24.3	21.9	20.7 to 23.2	21.0	19.8 to 22.2	20.1	19 to 21.3	19.5	18.4 to 20.7	22.5	22.1 to 22.9
France	14.5	14.2 to 14.8	14.3	14 to 14.6	13.9	13.6 to 14.2	12.1	11.9 to 12.4	12.3	12 to 12.5	13.7	13.6 to 13.8
Germany	39.3	38.9 to 39.7	39.6	39.2 to 40.1	37.6	37.2 to 38.1	34.5	34.1 to 34.9	33.0	32.6 to 33.4	36.7	36.6 to 36.9
Greece	26.6	25.6 to 27.5	24.3	23.4 to 25.2	25.5	24.6 to 26.4	23.6	22.8 to 24.5	25.2	24.3 to 26	25.6	25.3 to 25.9
Hungary	61.9	60.2 to 63.6	61.8	60.1 to 63.5	61.8	60.1 to 63.5	52.2	50.6 to 53.7	48.4	46.9 to 49.9	59.4	58.8 to 59.9
Ireland	50.4	47.8 to 53	51.5	48.9 to 54.1	52.4	49.9 to 55	42.8	40.6 to 45.1	40.1	37.9 to 42.2	52.4	51.5 to 53.2
Italy	31.8	31.4 to 32.2	29.1	28.7 to 29.5	29.3	28.9 to 29.7	28.4	28 to 28.8	24.2	23.8 to 24.5	28.8	28.7 to 29
Lithuania	26.5	24.4 to 28.7	22.9	21 to 24.9	19.3	17.5 to 21.1	19.1	17.3 to 20.8	15.1	13.6 to 16.7	24.9	24.2 to 25.5
Latvia	18.3	16.2 to 20.5	18.2	16.1 to 20.4	15.3	13.3 to 17.2	14.9	13 to 16.8	15.1	13.1 to 17	16.3	15.7 to 16.9
Luxembourg	39.3	33.3 to 45.3	40.5	34.5 to 46.5	42.7	36.6 to 48.8	32.9	27.6 to 38.2	32.9	27.7 to 38.1	36.7	34.9 to 38.5
Netherlands	46.6	45.5 to 47.8	45.8	44.7 to 46.9	43.5	42.5 to 44.5	35.0	34 to 35.9	34.2	33.3 to 35.1	44.9	44.6 to 45.3
Norway	53.8	51.6 to 56.1	52.2	50 to 54.3	50.6	48.5 to 52.7	45.6	43.6 to 47.6	48.1	46 to 50.1	51.2	50.5 to 51.9
Poland	23.4	22.8 to 23.9	21.6	21 to 22.1	19.7	19.1 to 20.2	19.7	19.1 to 20.2	16.4	15.9 to 16.8	22.8	22.6 to 22.9
Portugal	21.0	20.1 to 21.9	22.9	22 to 23.8	20.6	19.7 to 21.4	18.9	18.1 to 19.7	16.8	16 to 17.5	21.2	21 to 21.5
Slovenia	25.9	23.5 to 28.2	22.0	19.8 to 24.1	27.4	25.1 to 29.8	17.9	16 to 19.8	14.3	12.6 to 15.9	22.8	22.1 to 23.5
Slovakia	22.7	21.1 to 24.3	21.8	20.2 to 23.3	19.1	17.7 to 20.5	18.3	16.9 to 19.7	16.1	14.8 to 17.4	22.9	22.4 to 23.3
Spain	27.7	27.3 to 28.2	25.7	25.3 to 26.2	23.9	23.4 to 24.3	21.4	21 to 21.8	19.2	18.8 to 19.5	27.5	27.4 to 27.7
Sweden	34.1	32.9 to 35.3	32.9	31.8 to 34.1	30.1	29 to 31.2	28.7	27.7 to 29.8	25.5	24.5 to 26.5	31.0	30.6 to 31.3
Switzerland	26.3	25.1 to 27.5	25.2	24.1 to 26.3	25.1	24 to 26.2	19.8	18.8 to 20.8	19.6	18.6 to 20.6	25.1	24.7 to 25.4
UK	56.7	56.1 to 57.3	54.7	54.1 to 55.3							57.1	56.9 to 57.3
All Europe	34.3	34.1 to 34.4	33.2	33 to 33.3	29.2	29.1 to 29.4	26.7	26.6 to 26.9	24.9	24.7 to 25	32.1	32 to 32.1

Death rates are represented as per 100 000 person-years.

COPD, chronic obstructive pulmonary disease.

**Table 2:** Standardised mortality rates from 2011 to 2021 (a: 2011–2016 and b: 2017–2021) for COPD across all populations in Europe stratified by age, sex and year

(a)	2011		2012		2013		2014		2015		2016	
	Standardised rate	95% CI	Standardised rate	95% CI	Standardised rate	95% CI	Standardised rate	95% CI	Standardised rate	95% CI	Standardised rate	95% CI
Total population	34.7	34.5 to 34.9	35.0	34.8 to 35.1	34.9	34.7 to 35	32.3	32.1 to 32.4	35.1	35 to 35.3	33.3	33.1 to 33.5
Sex												
Female	22.0	21.8 to 22.2	22.7	22.5 to 22.9	23.1	22.9 to 23.3	21.5	21.3 to 21.7	24.1	23.9 to 24.3	23.2	23 to 23.3
Male	54.7	54.3 to 55	54.3	53.9 to 54.7	53.3	53.0 to 53.7	49.2	48.9 to 49.5	52.5	52.1 to 52.7	50.3	49.9 to 50.6
Age group (years)												
<55	0.9	0.8 to 0.9	0.8	0.8 to 0.8	0.8	0.8 to 0.9	0.8	0.7 to 0.8	0.8	0.8 to 0.9	0.8	0.8 to 0.9
55–59	12.2	11.8 to 12.6	11.3	10.9 to 11.7	12.5	12.1 to 12.9	11.2	10.8 to 11.5	12.5	12.1 to 12.8	12.4	12 to 12.8
60–64	25.4	24.8 to 26	24.5	23.9 to 25	26.0	25.4 to 26.6	24.2	23.7 to 24.8	27.6	27 to 28.2	27.1	26.5 to 27.7
65–69	46.7	45.8 to 47.6	48.1	47.2 to 49	48.5	47.6 to 49.3	45.6	44.8 to 46.4	50.1	49.3 to 50.9	48.8	48 to 49.6
70–74	80.0	78.8 to 81.2	80.9	79.7 to 82.2	84.9	83.6 to 86.1	79.7	78.5 to 80.9	88.2	86.9 to 89.4	88.7	87.4 to 90
75–79	145.1	143.3 to 147	146.8	144.9 to 148.6	146.8	145 to 148.7	135.2	133.5 to 136.9	145.3	143.6 to 147.1	138.8	137.1 to 140.5
80–84	262.7	259.7 to 265.7	272.6	269.5 to 275.6	261.2	258.3 to 264.1	240.0	237.2 to 242.8	255.8	253 to 258.6	236.3	233.6 to 238.9
>85	540.0	534.6 to 545.4	535.1	530 to 540.2	522.8	517.9 to 520	482.1	477.6 to 486.6	519.6	515 to 524.1	478.0	473.7 to 482.2

(b)	2017		2018		2019		2020		2021		2011–2021	
	Standardised rate	95% CI	Standardised rate	95% CI	Standardised rate	95% CI	Standardised rate	95% CI	Standardised rate	95% CI	Standardised rate	95% CI
Total population	34.3	34.1 to 34.4	33.2	33 to 33.3	29.2	29.1 to 29.4	26.7	26.6 to 26.9	24.9	24.7 to 25	32.1	32 to 32.1
Sex												
Female	24.5	24.3 to 24.7	24.0	23.9 to 24.2	20.5	20.3 to 20.7	18.6	18.4 to 19.7	17.7	17.5 to 17.8	22.0	22 to 22.1
Male	49.6	49.3 to 49.9	47.4	47.4 to 47.7	42.8	42.5 to 43.1	39.4	39.1 to 39.7	36.1	35.8 to 36.3	47.8	47.7 to 47.9
Age group (years)												
<55	0.8	0.8 to 0.9	0.9	0.8 to 0.9	0.7	0.7 to 0.8	0.7	0.6 to 0.7	0.6	0.6 to 0.7	0.8	0.8 to 0.8
55–59	12.1	11.7 to 12.5	12.4	12 to 12.8	10.9	10.5 to 11.3	9.4	9.1 to 9.7	9.2	8.9 to 9.5	11.5	11.4 to 11.6
60–64	27.7	27.1 to 28.3	28.0	27.4 to 28.6	24.8	24.3 to 25.4	22.6	22 to 23.1	21.4	20.9 to 21.9	25.4	25.3 to 25.6
65–69	50.8	50 to 51.6	50.8	50 to 51.6	45.8	44.9 to 46.6	43.5	42.7 to 44.3	41.7	40.9 to 42.5	47.4	47.2 to 47.7
70–74	90.2	89 to 91.5	89.1	87.9 to 90.3	77.0	75.8 to 78.2	71.4	70.2 to 72.5	68.1	67 to 69.2	81.7	81.3 to 82
75–79	143.5	141.7 to 145.2	141.9	140.2 to 143.6	123.1	121.4 to 124.8	112.0	110.4 to 113.6	110.1	108.4 to 111.7	135.6	135.1 to 136.2
80–84	238.5	235.9 to 241.2	227.6	225 to 230.2	197.2	194.7 to 199.7	179.8	177.4 to 182.1	163.6	161.4 to 165.9	229.7	228.9 to 230.6
> 85	493.7	489.5 to 498	460.7	456.6 to 464.7	418.7	414.7 to 422.8	382.1	378.3 to 385.9	339.7	336.2 to 343.3	465.9	464.6 to 467.2

Mortality rates are represented per 100 000 person-years.

COPD, chronic obstructive pulmonary disease.

**Figure 1:**
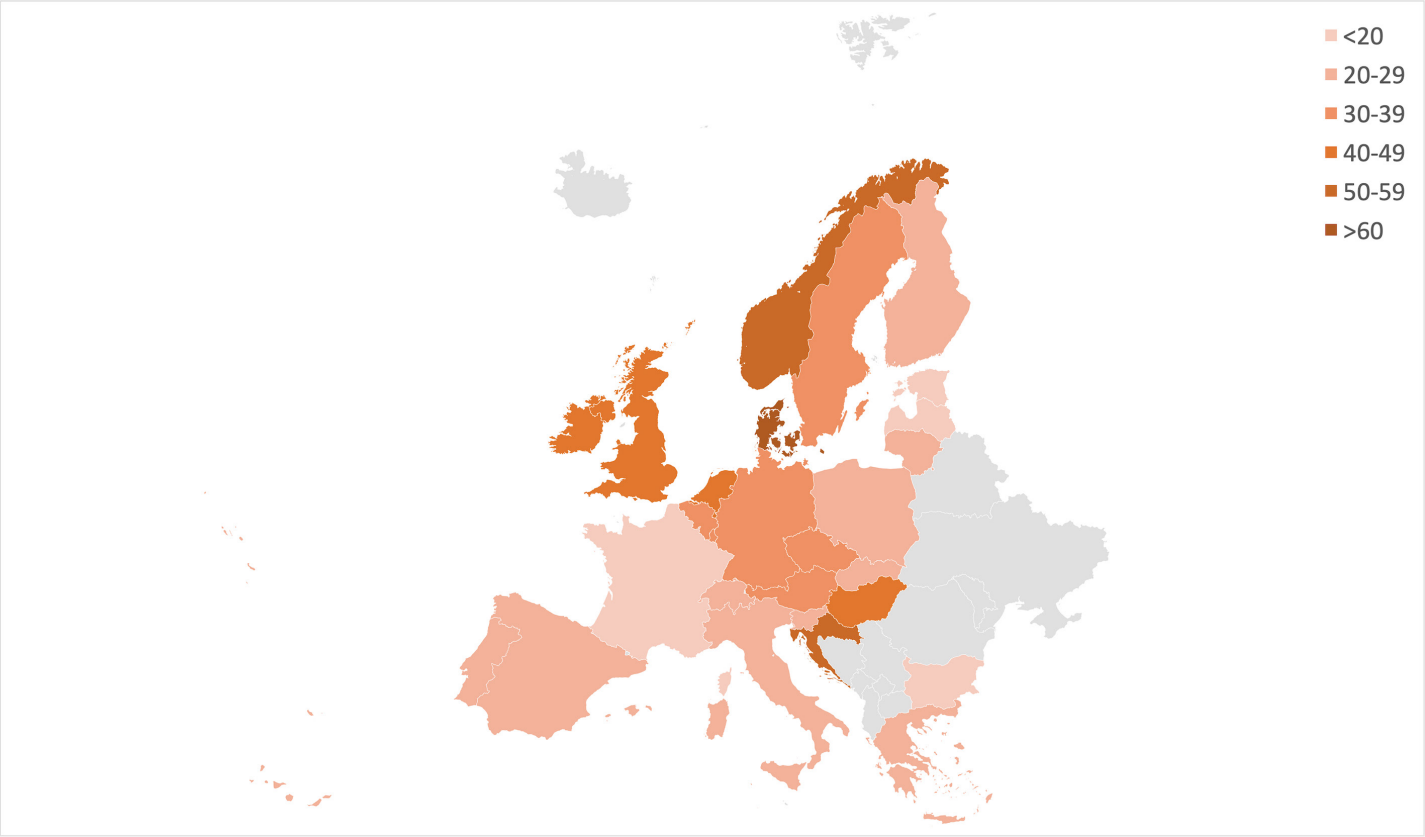
Geographical distribution of overall age-standardised and sex-standardised mortality rates for COPD from 2011 to 2021 across 27 European countries. Data are represented as mortality rates per 100 000 person-years. COPD, chronic obstructive pulmonary disease. The map is created by authors using the EUROSTAT data and GeoNames, Microsoft software.

Figure 1 shows the intercountry variation of overall mortality rates across Europe from 2011 to 2021. The countries with the largest death rates were Denmark (SMR 70.8 (95% CI 70.1 to 71.5) per 100 000 person-years), Hungary (SMR 59.4 (95% CI 58.8 to 59.9) per 100 000 person-years) and UK (SMR 57.1 (95% CI 56.9 to 57.3) per 100 000 person-years), respectively. There was a fivefold difference in mortality rates from 2011 to 2021 between the country with the highest rates (Denmark) versus the one with the least (France) (table 1a,b).

### Mortality trends 2011–2021

**Figure 2:**
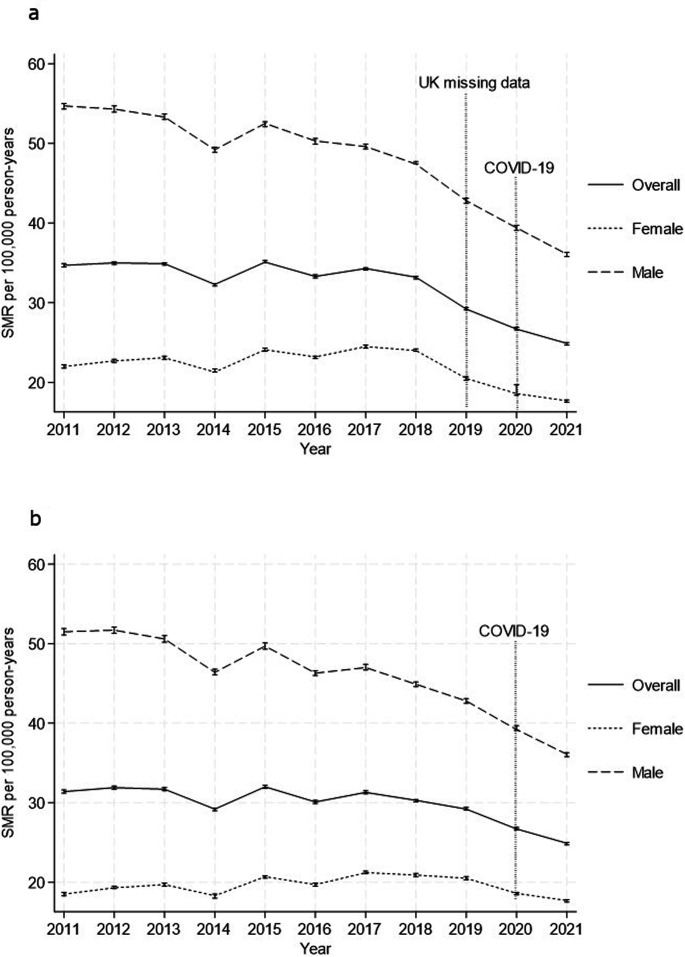
Overall age-standardised and sex-standardised mortality rates (solid line) and age-standardised mortality rates for males (dash) and females (dots) across Europe from 2011 to 2021 (**a**) with and (**b**) without the inclusion of UK mortality rates. The error bars are 95% CIs. SMR, standardised mortality rate.

Overall, from 2011 to 2021, there was a trend of reducing annual death rates within Europe (table 3 and figure 2a). However, the missing data from UK leaving the EUROSTAT database contributed to the steep drop in mortality from 2019 onwards, considering it was a high outlier. The COVID-19 pandemic may have also influenced the low death rates reported in 2020 and 2021 (figure 2a). The segmented regression analysis demonstrated no significant change in overall deaths between 2011 and 2018 (APC −0.7 (95% CI −1.8 to 0.8)%), followed by marked decline from 2019 (APC −9.9 (95% CI −16.3 to −6.4)%) (online supplemental figure S1a). Removal of all UK mortality data, during the sensitivity analysis, reduced the SMR across Europe to 29.6 (95% CI 29.6 to 29.7) per 100 000 person-years (online supplemental table S4). Furthermore, the overall annual mortality trend was similar to the main analysis, but the decline from 2019 and onwards was not as steep (online supplemental table S5, figure 2b and online supplemental figure S1b). There was also no significant change in deaths from 2011 to 2019 without UK data (AAPC: −0.7 (95% CI −1.7 to 0.4)%). The results of this sensitivity analysis, that is, mortality rates stratified by age and sex and the trends are available in online supplemental tables S4 and S5. The analysis of mortality rate trend for countries with more than 10 million population also demonstrated no significant change (online supplemental figure S2).

**Table 3:** Average annual percentage change in standardised mortality rates for COPD for the whole population in Europe as well as stratified by 27 European countries, sex and age over different time intervals

	AAPC 2011–2021 (%)	95% CI	AAPC 2011–2018 (%)	95% CI
Total population	−2.9*	−4.4 to (−1.6)	−0.5	−1.6 to 0.5
Country				
Austria	0.6	−0.7 to 1.9	1.4	−0.7 to 3.6
Belgium	−3.0*	−4.1 to (−1.5)	−1.9	−3.8 to 0.06
Bulgaria	−0.3	−5.5 to 5.4	−4.3*	−8.1 to (−0.5)
Czech Republic	1.6	−1.6 to 4.5	1.9	−2.2 to 6.2
Croatia	−7.6*	−13.6 to (−1.3)	4.4	−3.9 to 15.1
Denmark	−1.6*	−3.0 to (−0.3)	−0.4	−1.3 to 0.5
Estonia	−2.2*	−4.2 to (−0.3)	−1.2	−0.34 to 0.9
Finland	−1.9*	−2.3 to (−1.5)	−1.8*	−2.8 to (−0.8)
France	−1.3*	−2.3 to (−0.2)	0.7	−0.7 to 2.2
Germany	−0.8*	−1.49 to 0.0	1.2	−0.4 to 2.9
Greece	8.5	−22.61 to 55.6	−2.0	−11.6 to 8.0
Hungary	−14.1*	−22.5 to (−4.8)	1.0	−2.6 to 5.6
Ireland	−3.9*	−4.7 to (−3.0)	−2.2	−4.5 to 0.4
Italy	−1.4	−3.4 to 1.5	0.2	−1.9 to 2.3
Lithuania	−6.8*	−9.7 to (−4.3)	−4.5*	−8.9 to (−0.2)
Latvia	−0.3	−2.6 to 2.4	2.2	−0.03 to 4.7
Luxembourg	−0.7	−6.5 to 5.5	0.5	−3.6 to 4.8
Netherlands	−3.8*	−5.6 to (−1.9)	−2.1	−4.2 to 0.13
Norway	−1.1*	−1.8 to (−0.2)	0.1	−1.0 to 1.2
Poland	−4.7*	−8.4 to (−0.8)	−3.9*	−6.2 to (−1.6)
Portugal	−2.6*	−3.7 to (−1.5)	0.0	−2.7 to 2.8
Slovenia	−5.2	−9.9 to 0.9	−2.7	−6.2 to 0.9
Slovakia	−4.8	−9.4 to 0.0	−4.2*	−7.3 to (−1.1)
Spain	−5.3*	−6.8 to (−4.0)	−3.9*	−5.4 to (−2.4)
Sweden	−2.0*	−2.6 to (−1.3)	0.8	−0.5 to 2.1
Switzerland	−3.7*	−5.4 to (−1.8)	−1.1	−2.7 to 0.5
UK	−0.9	−2.4 to 0.7	−1.0	−2.4 to 0.6
Sex				
Female	−2.2*	−3.7 to (−0.8)	1.3*	0.1 to 2.6
Male	−3.8*	−5.2 to 2.5	−2.0^*^	−2.6 to (−1.2)
Age (years)				
<55	−3.0*	−5.6 to (−0.5)	0.1	−1.8 to 1.8
55–59	−2.5*	−4.3 to (−0.8)	0.6	−1.1 to 2.4
60–64	−1.3	−2.6 to 0.01	1.9*	0.5 to 3.4
65–69	−1.1	−2.5 to 0.4	1.2*	0.2 to 2.3
70–74	−1.7	−3.5 to 0.05	1.8*	0.8 to 2.9
75–79	−2.8*	−4.4 to (−1.3)	−0.4	−1.4 to 0.6
80–84	−4.7*	−6.4 to (−3.2)	−2.3*	−3.9 to (−0.5)
> 85	−4.1*	−5.2 to (−3.1)	−2.1*	−3.7 to (−1.4)

* p<0.05.

AAPC, average annual percentage change; COPD, chronic obstructive pulmonary disease.

### Mortality trends 2011–2018 stratified by country, age and sex

Given the above, when the Joinpoint regression analysis focused on calendar years 2011–2018, there was a 2% decrease in average annual deaths in males across all of Europe, while the rates increased in females (table 3). The death rates for ages 60–64, 65–69 and 70–74 years also rose year on year for this time frame in Europe (table 3).

When stratified by country, only six reported a statistically significant decline in average annual mortality (Bulgaria, Finland, Lithuania, Poland, Slovakia and Spain) and Latvia in fact displayed a 2% increment (table 3). The collated graphs representing SMR by calendar year for each individual country are available in online supplemental figure S3.

### Prevalence of current smokers

The average prevalence of current smoking tobacco use across Europe from 2011 to 2019 was 28 (95% CI 27 to 29)% and ranged from 14% up to 40% (online supplemental table S6). Smoking prevalence was lowest in the UK and Denmark (these were also the countries with the largest SMR in Europe). Conversely, Greece, Latvia and France had the greatest current smoking prevalence within Europe and recorded relatively modest changes from 1990 to 2019 (online supplemental tables S6 and S7).

## Discussion

Here, up-to-date insight into the burden of COPD-related mortality across 27 countries in Europe is provided. The plateauing of mortality rates from 2011 to 2018 is a concerning observation. In fact, mortality rates were on the rise in females. This is in the context marked heterogeneity in current tobacco smoking prevalence, which has major implications for policy making and public health.

The study focused on mortality trends between 2011 and 2018 across Europe because of missing UK data (from when they left the database), which was one of the high outliers, and due to the COVID-19 pandemic; both likely skewed the data 2019–2021. Reassuringly, the regression analysis from 2011 to 2019 without UK data also revealed no significant change in average annual mortality rates across the whole of Europe.

Though contemporary data on worldwide COPD mortality trends are available,^9^ few studies have focused on recent trajectories in Europe. Countries such as Belgium, Denmark, Slovenia, UK, France, Italy and Ireland that demonstrated steep improvements in mortality rates from 1994 to 2010^5^ had reduced AAPC in this study from 2011 to 2018, which is in line with the trend estimates documented by Marshall *et al* and Mei *et al*.^7 9^ The overall SMRs reported in the previous studies were lower than evidenced here.^7^,^9^ However, the variation is likely due to the differing methodologies for capturing mortality data in the relevant databases. GBD and WHO datasets rely on statistical modelling, which produces aggregate estimates.^13 14^ EUROSTAT, on the other hand, records directly reported national death registration data that is standardised across countries, enabling more reliable assessment of mortality trajectories and intercountry heterogeneity.^10^ An upward trending prevalence of COPD demonstrated in many observational studies^7 15^ is likely to translate to more COPD-related deaths, which may explain the plateauing mortality trajectories. The rising number of people with COPD may also be saturating the health service with an ever-growing number of patients with complex COPD-related care needs.^3 16^ Moreover, despite the successes of population-level smoking cessation strategies, the prevalence of current smoking tobacco use still remains high with marked geographical variability.^17 18^ In this study, the current smoking prevalence in many countries was upwards of 30%. Similarly, COPD care is still in need of new major disease-modifying interventions. The global initiative for chronic obstructive lung disease guidance has changed to a phenotypic-driven approach to managing COPD, such as addressing frequent exacerbations, breathlessness and comorbidities.^19^ However, this has often failed to be implemented in the real-world setting.^20^

Sustained decline in mortality rates in some countries was likely to be due to significant developments in the infrastructure for providing COPD-related care and continued successes of antismoking policies. For example, in Poland, there was a 54% reduction in annual hospital admissions due to COPD between 2006 and 2019, which was attributed to improvements in outpatient care.^21^ Furthermore, Moryson *et al* demonstrated improvements in premature mortality due to COPD in Poland from 2008 to 2017 owing to systemic actions taken to control tobacco dependence over the last three decades.^22^ Certainly, the socioeconomic growth in such Eastern European countries is likely to play a role in better access to healthcare and implementation of public health policies.^23^

The variation in mortality rates in different countries observed here may be attributed to a number of factors such as differences in local public health measures and guidance for the management of COPD.^24^ However, the validity and comparability of death certificates among different EU countries is also a likely major contributor.^25^ Many studies have suggested that COPD is often under-reported on most European death certificates, which could lead to underestimation of the mortality burden.^25^,^28^ Given the complex interplay between COPD and other comorbidities, it is understandable that the disease may not be listed as the underlying cause of death despite being a major contributor. As such, variability in listing COPD as the underlying cause of death may not reflect the real burden of COPD at a population level. COPD may also be underdiagnosed in some countries, which may then result in underestimation of related deaths.^29^ It is evident that access to spirometry, which is the gold standard for diagnosing COPD, is heterogeneous geographically and the diagnostic criteria for airway obstruction also varies between countries.^24 30^ In the UK, national incentives were employed, such as advocating spirometry to diagnose suspected COPD as part of the Quality and Outcomes Framework for primary care.^31^ This is likely to explain the high prevalence and therefore mortality observed in the UK.^32^ Similarly, a nationwide register for COPD cases was set up in Denmark from 2008, aiming to describe quality of hospital and community-based care provided for all patients diagnosed with COPD.^33^ In 2014, the database contained roughly 38 000 records with 91% of patients having spirometry measures documented and 97% offered participation in pulmonary rehabilitation if appropriate. It is therefore unlikely that the high mortality rates in Denmark reflect deficiencies in access to treatment. Conversely, countries of low prevalence are likely to reflect underdiagnoses of COPD and therefore may not be a true reflection of their underlying mortality burden. In a longitudinal population-based study, Lindberg *et al* highlighted only 28% of patients with COPD who died had COPD recorded within the death certificate and that under-recognition of COPD by clinicians contributed to the under-reporting.^29^

The gender gap in COPD-related mortality likely reflects a greater smoking exposure and burden of comorbidities in men.^34^ Although previously reported, the continued narrowing of the gender gap observed here is of importance.^5 9 35 36^ It may be explained by the stages of the tobacco epidemic model that males and females currently fall into.^37^ According to Lopez *et al*, there are different stages to the smoking epidemic; an initial incline in smoking prevalence followed by a further rapid increase, then a plateau and decrease, and finally a sustained reduction. Due to the delayed impact of smoking on health-related outcomes, the effects of tobacco prevalence trends on related mortality are only apparent decades later. Smoking prevalence in women lagged behind men by roughly a decade in high-income countries, so it is likely that current mortality trends in females represent an earlier stage of the model compared with males.^37^ Moreover, COPD is disproportionately under-reported in women than men,^38^ possibly owing to clinical presentations that are less typical of COPD.^39^ Women may incur delays in diagnosis and suffer worse prognosis as a result. The narrowing trend in mortality between sexes also highlights that blanket population-based policies may be favouring men more than women. Anatomical and physiological differences between sexes may result in greater susceptibility for disease progression in women with COPD and warrant more targeted therapeutic strategies. Heightened local inflammatory response in the airways and more pronounced small airway narrowing have been observed in female smokers compared with males.^40^ These are likely to accelerate the development of severe COPD.

Geographical variation in smoking behaviours plays a major role in the heterogeneity of COPD-related deaths observed in Europe, given it is the main aetiological factor for disease in high-income regions.^8^ In this study, countries such as Latvia, France and Greece reported a smoking prevalence of up to 40% and relatively modest decline from 1990 to 2019. Conversely, the SMR was relatively small in the same countries. Though the deleterious effects of tobacco burden were not reflected in contemporary mortality estimates, owing to the delayed smoking-related complications and deaths from other smoking-related comorbidities,^41 42^ this data provide insight to where targeted smoking cessation strategies can be employed.

The main strengths of this study are that it provides a more contemporaneous estimates and patterns of mortality across a wide range of European countries, using a validated and established database. The current work utilises ICD codes selective for COPD and emphysema, thus the mortality rates more confidently reflect the stratum of deceased people with COPD rather than other disease processes. In contrast, other studies assessing mortality trends a decade ago have included broader range of diagnoses, which includes chronic bronchitis and bronchiectasis clumped together. The subanalysis of trends in countries with large population sizes yielded similar findings to the main findings, which further strengthen the validity of the results.

There are also some limitations to mention; first, the general challenges with accuracy of death certificates mean that mortality is likely under-reported, though this is an issue with many studies reporting COPD-related mortality. Specifically, EUROSTAT records primary cause of death data, which means COPD as a contributory cause or other comorbidities are not captured. However, it is reassuring that the trends and the gender gap narrowing reported here are supported by previous studies. The impact of missing data due to UK leaving the EUROSTAT dataset and the pandemic confounded the interpretation of standard mortality statistics for the entire time interval of analysis. Moreover, we only had surrogate measures for smoking burden, though it should be noted this was not the main focus of the study analysis.

In conclusion, the apparent plateauing of COPD-related mortality rates across Europe is a major concern and cannot be ignored. The gender gap narrowing demands further focus, and the intercountry heterogeneity in deaths poses a real question on the true extent of COPD burden. These data support the need for ongoing inter-European public health initiatives to improve disease burden.

## Supplementary material

10.1136/bmjresp-2025-003175 online supplemental file 1

## Data Availability

Data are available in a public, open access repository.
