# Supplementary material for: Mortality trends in chronic obstructive pulmonary disease in 27 countries within Europe from 2011 to 2021
Source: BMJ Open Respir Res. 2026 Jan 5;13(1):e003175. doi: 10.1136/bmjresp-2025-003175 (PMC12778345; doi:10.1136/bmjresp-2025-003175)
Supplement: online supplemental file 1 [file bmjresp-13-1-s001.docx]

**Supplementary material**

[Table S1. 2](#_Toc187613193)

[Table S2. 3](#_Toc187613194)

[Table S3. 4](#_Toc187613195)

[Table S4. 6](#_Toc187613196)

[Table S5. 7](#_Toc187613197)

[Table S6. 8](#_Toc187613198)

[Table S7. 9](#_Toc187613199)

[Figure S1…………………………………………………………………………………………………………………………….10](file:///C:\Users\mszag4.AD\Documents\ACL\eurostat%20sup%20data_updated151024.docx#_Toc187613253)

[Figure S2.. 11](#_Toc187613254)

[Figure S3. 14](file:///C:\Users\mszag4.AD\Documents\ACL\eurostat%20sup%20data_updated151024.docx#_Toc187613255)

Table S1. Overall population in Europe in 2018 (reference population) stratified by age and sex

|  | Standard population | | | |
| --- | --- | --- | --- | --- |
|  | Female | | Male | |
|  | (n) | (%) | (n) | (%) |
| Total population | 251943469 | . | 241018749 | . |
| Age group (years) |  |  |  |  |
| <55 | 161496667 | 64.10 | 165765135 | 68.78 |
| 55-59 | 17623105 | 6.99 | 16998387 | 7.05 |
| 60-64 | 16257551 | 6.45 | 15135471 | 6.28 |
| 65-69 | 15131687 | 6.01 | 13561780 | 5.63 |
| 70-74 | 12670148 | 5.03 | 10825921 | 4.49 |
| 75-79 | 10833985 | 4.30 | 8416900 | 3.49 |
| 80-84 | 8713708 | 3.46 | 5865252 | 2.43 |
| > 85 | 9216618 | 3.66 | 4449903 | 1.85 |

Table S2. Raw deaths related to COPD and emphysema codes and corresponding population across all European countries stratified by age, sex and years

|  | **2011** | | | **2012** | | | **2013** | | | **2014** | | | **2015** | | | **2016** | | | **2017** | | | **2018** | | | **2019** | | | **2020** | | | **2021** | | |  |
| --- | --- | --- | --- | --- | --- | --- | --- | --- | --- | --- | --- | --- | --- | --- | --- | --- | --- | --- | --- | --- | --- | --- | --- | --- | --- | --- | --- | --- | --- | --- | --- | --- | --- | --- |
|  | Raw deaths | | Population | Raw deaths | | Population | Raw deaths | | Population | Raw deaths | | Population | Raw deaths | | Population | Raw deaths | | Population | Raw deaths | | Population | Raw deaths | | Population | Raw deaths | | Population | Raw deaths | | Population | Raw deaths | | Population | |
|  | J43 | J44 |  | J43 | J44 |  | J43 | J44 |  | J43 | J44 |  | J43 | J44 |  | J43 | J44 |  | J43 | J44 |  | J43 | J44 |  | J43 | J44 |  | J43 | J44 |  | J43 | J44 |  | |
|  | (n) | (n) | (n) | (n) | (n) | (n) | (n) | (n) | (n) | (n) | (n) | (n) | (n) | (n) | (n) | (n) | (n) | (n) | (n) | (n) | (n) | (n) | (n) | (n) | (n) | (n) | (n) | (n) | (n) | (n) | (n) | (n) | (n) | |
| **Total population** | 7062 | 131294 | 481134264 | 7255 | 138141 | 483029543 | 7619 | 141319 | 484218001 | 7302 | 138131 | 486568773 | 8059 | 154316 | 488144303 | 7945 | 149328 | 490096287 | 8085 | 157609 | 491570175 | 8440 | 155063 | 492962218 | 7142 | 121820 | 493924507 | 6743 | 113735 | 430107010 | 6896 | 106612 | 429313915 | |
| **Sex** |  |  |  |  |  |  |  |  |  |  |  |  |  |  |  |  |  |  |  |  |  |  |  |  |  |  |  |  |  |  |  |  |  | |
| **Female** | 2390 | 52511 | 246516847 | 2478 | 56367 | 247541978 | 2602 | 58356 | 248082490 | 2470 | 56833 | 249206072 | 2697 | 64982 | 249922336 | 2627 | 63200 | 250651269 | 2713 | 68252 | 251314871 | 2914 | 67481 | 251943469 | 2519 | 51525 | 252357534 | 2255 | 47529 | 219981170 | 2313 | 45230 | 219516125 | |
| **Male** | 4672 | 78783 | 234617417 | 4777 | 81774 | 235487565 | 5017 | 82963 | 236135511 | 4832 | 81298 | 237362701 | 5362 | 89334 | 238221967 | 5318 | 86128 | 239445018 | 5372 | 89357 | 240255304 | 5526 | 87582 | 241018749 | 4623 | 70295 | 241566973 | 4488 | 66206 | 210125840 | 4583 | 61382 | 209797790 | |
| **Age group (years)** |  |  |  |  |  |  |  |  |  |  |  |  |  |  |  |  |  |  |  |  |  |  |  |  |  |  |  |  |  |  |  |  |  | |
| **<55** | 295 | 2502 | 333192506 | 232 | 2305 | 332269782 | 280 | 2388 | 331094910 | 272 | 2318 | 330711232 | 256 | 2559 | 329764218 | 289 | 2426 | 329336250 | 280 | 2477 | 328276219 | 302 | 2574 | 327261802 | 183 | 1889 | 325815858 | 169 | 1662 | 279963093 | 175 | 1592 | 277546777 | |
| **55-59** | 303 | 3409 | 31654303 | 269 | 3253 | 32054290 | 326 | 3646 | 32440215 | 277 | 3406 | 32930150 | 352 | 3797 | 33381013 | 359 | 3835 | 33818470 | 314 | 3836 | 34234537 | 335 | 3951 | 34621492 | 290 | 3058 | 35102369 | 224 | 2714 | 31193771 | 263 | 2629 | 31417785 | |
| **60-64** | 502 | 6757 | 29885800 | 501 | 6640 | 30124817 | 607 | 7044 | 30207985 | 510 | 6791 | 30259446 | 603 | 7753 | 30396845 | 569 | 7708 | 30619206 | 596 | 7963 | 31009182 | 669 | 8093 | 31393022 | 524 | 6427 | 31792448 | 479 | 5924 | 28444281 | 518 | 5610 | 28711957 | |
| **65-69** | 673 | 9884 | 23545716 | 721 | 10381 | 24294612 | 797 | 11122 | 25400477 | 821 | 11113 | 26409927 | 913 | 12643 | 27292395 | 843 | 12897 | 28383626 | 917 | 13510 | 28620325 | 977 | 13489 | 28693467 | 844 | 10611 | 28671421 | 767 | 10183 | 25446647 | 756 | 9818 | 25607797 | |
| **70-74** | 921 | 15637 | 21866923 | 1011 | 15812 | 21882237 | 1014 | 16661 | 21834749 | 976 | 16138 | 21940239 | 1139 | 17877 | 22004293 | 1157 | 17779 | 21741462 | 1128 | 18803 | 22462973 | 1204 | 19422 | 23496069 | 1010 | 14942 | 24383278 | 1026 | 14277 | 21864825 | 1056 | 14203 | 22800453 | |
| **75-79** | 1144 | 22330 | 17685252 | 1145 | 23028 | 17914478 | 1190 | 23471 | 18173659 | 1224 | 22710 | 18586564 | 1261 | 25030 | 18893718 | 1320 | 24301 | 19217032 | 1373 | 25293 | 19279305 | 1369 | 25018 | 19250885 | 1257 | 18925 | 19315058 | 1098 | 17307 | 17024407 | 1200 | 16541 | 16666116 | |
| **80-84** | 1355 | 28711 | 13138836 | 1412 | 30551 | 13333622 | 1360 | 29924 | 13537866 | 1352 | 28604 | 13653531 | 1412 | 31408 | 13890001 | 1344 | 29355 | 14044320 | 1352 | 30464 | 14323598 | 1379 | 29698 | 14578960 | 1178 | 23055 | 14912957 | 1154 | 21417 | 13490481 | 1185 | 19836 | 13726299 | |
| **> 85** | 1869 | 42064 | 10164928 | 1964 | 46171 | 11155705 | 2045 | 47063 | 11528140 | 1870 | 47051 | 12077684 | 2123 | 53249 | 12521820 | 2064 | 51027 | 12935921 | 2125 | 55263 | 13364036 | 2205 | 52818 | 13666521 | 1856 | 42913 | 13931118 | 1826 | 40251 | 12679505 | 1743 | 36383 | 12836731 | |

Table S3. Crude COPD related death rates stratified by 27 countries across Europe and by years. Death rates are represented as per 100,000 person-years. CI: confidence interval

|  | **2011** | | | **2012** | | | **2013** | | | **2014** | | | **2015** | | | **2016** | | | **2017** | | | | **2018** | | | | **2019** | | | | **2020** | | | | **2021** | | | | **2011-2021** | | |
| --- | --- | --- | --- | --- | --- | --- | --- | --- | --- | --- | --- | --- | --- | --- | --- | --- | --- | --- | --- | --- | --- | --- | --- | --- | --- | --- | --- | --- | --- | --- | --- | --- | --- | --- | --- | --- | --- | --- | --- | --- | --- |
| **Country** | **Deaths** | **Crude rate** | **95% CI** | **Deaths** | **Crude rate** | **95% CI** | **Deaths** | **Crude rate** | **95% CI** | **Deaths** | **Crude rate** | **95% CI** | **Deaths** | **Crude rate** | **95% CI** | **Deaths** | **Crude rate** | **95% CI** | **Deaths** | **Crude rate** | **95% CI** | **Deaths** | | **Crude rate** | **95% CI** | **Deaths** | | **Crude rate** | **95% CI** | **Deaths** | | **Crude rate** | **95% CI** | **Deaths** | | **Crude rate** | **95% CI** | **Deaths** | | **Crude rate** | **95% CI** |
|  | **(n)** |  |  | **(n)** |  |  | **(n)** |  |  | **(n)** |  |  | **(n)** |  |  | **(n)** |  |  | **(n)** |  |  | **(n)** | |  |  | **(n)** | |  |  | **(n)** | |  |  | **(n)** | |  |  | **(n)** | |  |  |
| **Austria** | 2299 | **28.18** | 27.04-29.35 | 2385 | **29.12** | 27.96-30.31 | 2545 | **30.92** | 29.73-32.15 | 2415 | **29.15** | 28-30.34 | 2749 | **32.88** | 31.66-34.14 | 2645 | **31.22** | 30.04-32.43 | 2954 | **34.58** | 33.34-35.85 | 2932 | | **34.13** | 32.9-35.38 | 3004 | | **34.79** | 33.56-36.06 | 2958 | | **34.06** | 32.84-35.31 | 2674 | | **30.66** | 29.51-31.85 | 29560 | | **31.83** | 31.47-32.19 |
| **Belgium** | 3935 | **36.76** | 35.62-37.92 | 4264 | **39.55** | 38.37-40.75 | 4223 | **38.95** | 37.78-40.14 | 3824 | **35.13** | 34.03-36.26 | 4148 | **37.90** | 36.75-39.07 | 3981 | **36.11** | 34.99-37.25 | 4080 | **36.85** | 35.73-38 | 4148 | | **37.30** | 36.17-38.45 | 4139 | | **37.03** | 35.91-38.17 | 3754 | | **33.39** | 32.33-34.47 | 3544 | | **31.42** | 30.39-32.47 | 44040 | | **36.37** | 36.03-36.71 |
| **Bulgaria** | . | **.** | . | . | **.** | . | 1274 | **17.89** | 16.92-18.9 | 1255 | **17.72** | 16.75-18.73 | 1395 | **19.81** | 18.78-20.88 | 1144 | **16.35** | 15.42-17.33 | 1089 | **15.68** | 14.76-16.64 | 1122 | | **16.30** | 15.36-17.29 | 1305 | | **19.10** | 18.08-20.17 | 1427 | | **21.03** | 19.95-22.15 | 1275 | | **18.87** | 17.85-19.94 | 11365 | | **16.85** | 16.54-17.16 |
| **Czech Republic** | 2319 | **22.65** | 21.73-23.59 | 2261 | **22.02** | 21.13-22.95 | 3201 | **31.19** | 30.12-32.29 | 2749 | **26.80** | 25.81-27.82 | 3332 | **32.40** | 31.3-33.51 | 2965 | **28.77** | 27.74-29.83 | 3246 | **31.42** | 30.35-32.52 | 3227 | | **31.15** | 30.08-32.24 | 3182 | | **30.61** | 29.56-31.7 | 3026 | | **29.00** | 27.98-30.06 | 2931 | | **28.64** | 27.61-29.69 | 32439 | | **28.61** | 28.3-28.93 |
| **Croatia** | 1503 | **35.93** | 34.14-37.8 | 1561 | **29.08** | 27.45-30.78 | 1623 | **30.24** | 28.58-31.97 | 1661 | **40.05** | 38.14-42.02 | 1864 | **45.20** | 43.17-47.3 | 1677 | **40.97** | 39.03-42.98 | 1848 | **45.52** | 43.47-47.65 | 1766 | | **44.00** | 41.97-46.1 | 1841 | | **46.19** | 44.1-48.35 | 1595 | | **40.17** | 38.22-42.19 | 1535 | | **38.89** | 36.97-40.89 | 18474 | | **39.62** | 39.04-40.21 |
| **Denmark** | 3213 | **59.28** | 57.25-61.37 | 3294 | **60.53** | 58.48-62.63 | 3366 | **61.64** | 59.58-63.76 | 3260 | **59.43** | 57.41-61.5 | 3335 | **60.47** | 58.44-62.56 | 3397 | **61.12** | 59.08-63.21 | 3586 | **64.01** | 61.93-66.14 | 3709 | | **65.76** | 63.66-67.91 | 3474 | | **61.30** | 59.28-63.37 | 3300 | | **58.06** | 56.09-60.07 | 3572 | | **62.69** | 60.65-64.78 | 37506 | | **61.31** | 60.69-61.94 |
| **Estonia** | 173 | **13.37** | 11.45-15.51 | 191 | **14.79** | 12.76-17.04 | 190 | **23.54** | 20.31-27.14 | 208 | **24.32** | 21.13-27.86 | 195 | **22.74** | 19.66-26.16 | 202 | **23.44** | 20.32-26.91 | 190 | **14.78** | 12.76-17.04 | 197 | | **51.28** | 44.37-58.96 | 165 | | **13.25** | 11.3-15.43 | 159 | | **18.07** | 15.37-21.1 | 176 | | **19.94** | 17.1-23.11 | 2046 | | **19.22** | 18.4-20.07 |
| **Finland** | 1143 | **21.82** | 20.57-23.12 | 1151 | **21.86** | 20.62-23.16 | 1127 | **31.54** | 29.72-33.43 | 1136 | **21.37** | 20.14-22.65 | 1201 | **22.49** | 21.24-23.8 | 1189 | **22.19** | 20.95-23.49 | 1235 | **22.97** | 21.71-24.29 | 1207 | | **22.39** | 21.15-23.69 | 1189 | | **22.01** | 20.77-23.29 | 1175 | | **21.70** | 20.48-22.98 | 1174 | | **21.65** | 20.43-22.92 | 12927 | | **22.64** | 22.25-23.03 |
| **France** | 7683 | **12.14** | 11.87-12.42 | 8310 | **13.07** | 12.79-13.35 | 8673 | **13.58** | 13.29-13.87 | 8133 | **12.62** | 12.35-12.9 | 9330 | **14.41** | 14.12-14.71 | 9247 | **14.24** | 13.95-14.53 | 9668 | **14.84** | 14.54-15.13 | 9650 | | **14.76** | 14.46-15.05 | 9592 | | **14.63** | 14.34-14.93 | 8516 | | **12.96** | 12.69-13.24 | 8649 | | **13.10** | 12.83-13.38 | 97451 | | **13.67** | 13.59-13.76 |
| **Germany** | 27287 | **34.90** | 34.49-35.32 | 28027 | **35.80** | 35.38-36.22 | 30464 | **38.85** | 38.41-39.29 | 28550 | **36.30** | 35.88-36.73 | 32860 | **41.56** | 41.11-42.01 | 31556 | **39.44** | 39-39.87 | 34010 | **42.32** | 41.87-42.77 | 34829 | | **43.18** | 42.72-43.63 | 33586 | | **41.49** | 41.04-41.93 | 31446 | | **38.73** | 38.3-39.16 | 30556 | | **37.62** | 37.2-38.04 | 343171 | | **39.13** | 39-39.26 |
| **Greece** | . | **.** | . | . | **.** | . | . | **.** | . | 2790 | **26.13** | 25.17-27.12 | 3443 | **32.46** | 31.39-33.57 | 2945 | **27.98** | 26.98-29.01 | 3232 | **30.78** | 29.72-31.86 | 3004 | | **28.67** | 27.65-29.71 | 3220 | | **30.77** | 29.72-31.86 | 3018 | | **28.86** | 27.84-29.9 | 3251 | | **31.20** | 30.14-32.29 | 24903 | | **29.60** | 29.23-29.97 |
| **Hungary** | 3880 | **33.32** | 32.18-34.5 | 4216 | **43.53** | 42.23-44.86 | 4473 | **46.34** | 44.99-47.71 | 4537 | **46.93** | 45.58-48.32 | 5467 | **56.71** | 55.22-58.23 | 4783 | **49.76** | 48.36-51.19 | 5206 | **54.43** | 52.97-55.93 | 5212 | | **54.64** | 53.16-56.14 | 5297 | | **55.56** | 54.07-57.07 | 4552 | | **47.76** | 46.38-49.17 | 4244 | | **44.71** | 43.37-46.07 | 51867 | | **48.51** | 48.09-48.93 |
| **Ireland** | 1372 | **30.92** | 29.3-32.6 | 1485 | **33.32** | 31.65-35.06 | 1518 | **33.89** | 32.21-35.64 | 1435 | **31.83** | 30.2-33.52 | 1552 | **34.11** | 32.43-35.85 | 1566 | **34.03** | 32.37-35.76 | 1472 | **31.60** | 30.01-33.26 | 1575 | | **33.48** | 31.85-35.18 | 1651 | | **34.57** | 32.92-36.28 | 1409 | | **29.15** | 27.65-30.71 | 1371 | | **28.10** | 26.63-29.63 | 16406 | | **32.24** | 31.75-32.74 |
| **Italy** | 17925 | **30.95** | 30.5-31.41 | 18931 | **32.67** | 32.2-33.14 | 17878 | **30.71** | 30.26-31.16 | 17734 | **29.93** | 29.5-30.38 | 19476 | **32.85** | 32.39-33.32 | 20697 | **34.98** | 34.5-35.46 | 22538 | **38.12** | 37.63-38.63 | 20989 | | **35.56** | 35.08-36.04 | 21435 | | **36.71** | 36.22-37.2 | 21259 | | **36.49** | 36-36.98 | 18228 | | **31.50** | 31.05-31.97 | 217090 | | **33.69** | 33.55-33.83 |
| **Lithuania** | 689 | **23.19** | 21.49-24.99 | 677 | **23.14** | 21.43-24.95 | 712 | **24.58** | 22.81-26.46 | 578 | **20.14** | 18.53-21.85 | 646 | **22.68** | 20.96-24.5 | 552 | **19.61** | 18.01-21.31 | 621 | **22.37** | 20.64-24.2 | 535 | | **19.53** | 17.91-21.26 | 456 | | **16.73** | 15.23-18.34 | 458 | | **16.80** | 15.3-18.41 | 360 | | **13.19** | 11.86-14.63 | 6284 | | **20.26** | 19.76-20.76 |
| **Latvia** | 240 | **11.86** | 10.41-13.46 | 239 | **11.98** | 10.51-13.6 | 252 | **12.76** | 11.23-14.44 | 240 | **12.29** | 10.78-13.95 | 221 | **11.88** | 10.37-13.56 | 292 | **15.21** | 13.51-17.05 | 293 | **15.41** | 13.69-17.27 | 291 | | **16.09** | 14.29-18.05 | 241 | | **12.86** | 11.28-14.58 | 241 | | **12.94** | 11.36-14.68 | 243 | | **19.24** | 16.9-21.82 | 2793 | | **13.67** | 13.16-14.18 |
| **Luxembourg** | 132 | **41.73** | 34.92-49.49 | 138 | **27.87** | 23.41-32.93 | 126 | **24.12** | 21.45-27.89 | 116 | **22.40** | 18.51-26.86 | 147 | **27.76** | 23.45-32.63 | 132 | **36.73** | 30.74-43.56 | 165 | **28.69** | 24.48-33.42 | 174 | | **46.14** | 39.54-53.53 | 189 | | **31.60** | 27.26-36.44 | 150 | | **38.06** | 32.21-44.66 | 153 | | **24.73** | 20.97-28.97 | 1622 | | **33.10** | 31.51-34.75 |
| **Netherlands** | 6335 | **39.07** | 38.11-40.04 | 6874 | **42.19** | 41.2-43.2 | 6526 | **39.93** | 38.97-40.91 | 5728 | **34.94** | 34.04-35.86 | 6897 | **41.89** | 40.9-42.89 | 6502 | **39.28** | 38.33-40.25 | 6807 | **40.87** | 39.91-41.85 | 6870 | | **41.00** | 40.04-41.98 | 6722 | | **39.90** | 38.96-40.87 | 5564 | | **32.78** | 31.92-33.65 | 5590 | | **32.78** | 31.92-33.65 | 70415 | | **38.57** | 38.29-38.86 |
| **Norway** | 1938 | **40.44** | 38.66-42.28 | 2078 | **42.78** | 40.96-44.65 | 2030 | **41.25** | 39.47-43.08 | 1954 | **39.26** | 37.53-41.04 | 2110 | **41.92** | 40.15-43.75 | 2157 | **42.46** | 40.69-44.29 | 2228 | **43.48** | 41.69-45.33 | 2203 | | **42.68** | 40.92-44.5 | 2189 | | **42.14** | 40.39-43.94 | 2024 | | **38.65** | 36.99-40.38 | 2187 | | **41.57** | 39.85-43.35 | 23098 | | **41.51** | 40.98-42.05 |
| **Poland** | 7077 | **19.06** | 18.62-19.51 | 6734 | **18.11** | 17.68-18.55 | 7142 | **19.20** | 18.76-19.65 | 5967 | **16.05** | 15.64-16.46 | 6684 | **17.98** | 17.55-18.42 | 6065 | **16.33** | 15.92-16.74 | 6728 | **18.11** | 17.68-18.55 | 6418 | | **17.29** | 16.87-17.72 | 5990 | | **16.13** | 15.73-16.55 | 6076 | | **16.37** | 15.96-16.79 | 5070 | | **13.99** | 13.61-14.38 | 69951 | | **17.15** | 17.03-17.28 |
| **Portugal** | 2016 | **19.54** | 18.7-20.41 | 2205 | **21.43** | 20.54-22.34 | 2021 | **19.73** | 18.88-20.61 | 2095 | **20.57** | 19.7-21.47 | 2311 | **22.80** | 21.88-23.75 | 2317 | **22.94** | 22.02-23.89 | 2198 | **21.83** | 20.93-22.76 | 2452 | | **24.39** | 23.44-25.38 | 2257 | | **22.49** | 21.57-23.43 | 2133 | | **21.22** | 20.33-22.14 | 1927 | | **19.17** | 18.32-20.05 | 23932 | | **21.46** | 21.19-21.73 |
| **Slovenia** | 426 | **21.34** | 19.36-23.47 | 402 | **31.10** | 28.13-34.29 | 359 | **26.69** | 24-29.6 | 366 | **18.22** | 16.4-20.18 | 393 | **28.93** | 26.14-31.94 | 378 | **56.13** | 50.62-62.09 | 469 | **23.28** | 21.22-25.48 | 407 | | **20.18** | 18.27-22.24 | 530 | | **26.08** | 23.91-28.4 | 354 | | **49.24** | 44.25-54.65 | 290 | | **20.29** | 18.02-22.76 | 4374 | | **25.90** | 25.14-26.68 |
| **Slovakia** | 899 | **17.09** | 15.99-18.25 | 998 | **18.94** | 17.78-20.15 | 729 | **13.81** | 12.82-14.85 | 777 | **14.70** | 13.69-15.78 | 884 | **16.71** | 15.62-17.84 | 844 | **15.93** | 14.87-17.04 | 827 | **15.58** | 14.54-16.68 | 824 | | **15.50** | 14.46-16.6 | 747 | | **14.04** | 13.05-15.09 | 730 | | **13.71** | 12.73-14.74 | 681 | | **12.78** | 11.84-13.78 | 8940 | | **15.34** | 15.02-15.66 |
| **Spain** | 13505 | **29.68** | 29.18-30.18 | 14417 | **31.58** | 31.07-32.1 | 13007 | **28.55** | 28.06-29.04 | 13167 | **29.04** | 28.55-29.54 | 14238 | **31.44** | 30.93-31.96 | 12669 | **27.98** | 27.49-28.47 | 13062 | **28.79** | 28.3-29.29 | 12336 | | **27.10** | 26.63-27.58 | 11658 | | **25.45** | 24.99-25.92 | 10688 | | **23.13** | 22.69-23.57 | 9638 | | **20.83** | 20.41-21.25 | 138385 | | **27.58** | 27.43-27.72 |
| **Sweden** | 2683 | **29.23** | 28.13-30.35 | 2844 | **30.76** | 29.64-31.91 | 2757 | **29.60** | 28.51-30.73 | 2727 | **29.04** | 27.96-30.15 | 2834 | **29.86** | 28.77-30.98 | 2988 | **31.14** | 30.04-32.28 | 3171 | **32.57** | 31.45-33.72 | 3123 | | **31.64** | 30.54-32.77 | 2907 | | **29.12** | 28.07-30.2 | 2852 | | **28.29** | 27.27-29.35 | 2571 | | **25.38** | 24.41-26.38 | 31457 | | **29.67** | 29.35-30 |
| **Switzerland** | 1741 | **22.71** | 21.65-23.8 | 1826 | **23.57** | 22.5-24.68 | 1944 | **24.84** | 23.75-25.97 | 1836 | **23.18** | 22.13-24.27 | 1989 | **24.81** | 23.73-25.92 | 1885 | **23.26** | 22.22-24.33 | 1989 | **24.26** | 23.21-25.35 | 1952 | | **23.62** | 22.58-24.69 | 1996 | | **23.97** | 22.93-25.04 | 1614 | | **19.23** | 18.3-20.19 | 1618 | | **19.13** | 18.21-20.08 | 20390 | | **22.93** | 22.62-23.25 |
| **UK** | 27901 | **45.84** | 45.3-46.38 | 29851 | **48.33** | 47.79-48.88 | 30778 | **49.52** | 48.96-50.07 | 30195 | **48.23** | 47.68-48.77 | 32674 | **51.77** | 51.21-52.33 | 32498 | **51.06** | 50.51-51.62 | 32782 | **51.12** | 50.57-51.68 | 32351 | | **50.11** | 49.56-50.66 | . | | **.** | **.** | . | | **.** | **.** | . | | **.** | **.** | 249030 | | **49.52** | 49.33-49.72 |
| **All Europe** | 138314 | **29.51** | 29.36-29.67 | 145359 | **31.14** | 30.98-31.3 | 148938 | **31.59** | 31.43-31.75 | 145433 | **29.92** | 29.76-30.07 | 162375 | **33.34** | 33.18-33.51 | 157273 | **32.22** | 32.06-32.38 | 165694 | **33.71** | 33.54-33.87 | 163503 | | **33.25** | 33.09-33.41 | 128962 | | **30.07** | 29.9-30.23 | 120478 | | **28.14** | 27.98-29.3 | 113508 | | **26.54** | 26.39-26.7 | 1589916 | | **30.95** | 30.91-31 |

Table S4. Age- and sex- standardised mortality rates from 2011–2021 without UK data, stratified by age and sex. Mortality rates are represented per 100,000 person-years. CI: confidence interval

|  | **2011** | | **2012** | | **2013** | | **2014** | | **2015** | | **2016** | | **2017** | | **2018** | | **2019** | | | **2020** | | | **2021** | | | **2011-2021** | |
| --- | --- | --- | --- | --- | --- | --- | --- | --- | --- | --- | --- | --- | --- | --- | --- | --- | --- | --- | --- | --- | --- | --- | --- | --- | --- | --- | --- |
|  | Standardised Rate | 95% CI | Standardised Rate | 95% CI | Standardised Rate | 95% CI | Standardised Rate | 95% CI | Standardised Rate | 95% CI | Standardised Rate | 95% CI | Standardised Rate | 95% CI | Standardised Rate | 95% CI | Standardised Rate | 95% CI | Standardised Rate | | 95% CI | Standardised Rate | | 95% CI | Standardised Rate | | 95% CI |
| **Total population** | **31.40** | 31.2-31.6 | **31.90** | 31.7-32.1 | **31.70** | 31.5-31.9 | **29.20** | 29-29.3 | **32.00** | 31.8-32.2 | **30.10** | 29.9-30.3 | **31.30** | 31.1-31.5 | **30.30** | 30.1-30.4 | **29.20** | 29.1-29.4 | **26.70** | | 26.6-26.9 | **24.90** | | 24.7-25 | **29.60** | | 29.6-29.7 |
| **Sex** |  |  |  |  |  |  |  |  |  |  |  |  |  |  |  |  |  |  |  | |  |  | |  |  | |  |
| **Female** | **18.50** | 18.3-18.7 | **19.30** | 19.2-19.5 | **19.70** | 19.5-19.9 | **18.30** | 18-18.5 | **20.70** | 20.5-20.8 | **19.70** | 19.5-19.9 | **21.20** | 21.1-21.4 | **20.90** | 20.7-21.1 | **20.50** | 20.3-20.7 | **18.60** | | 18.4-18.7 | **17.70** | | 17.5-17.8 | **19.50** | | 19.5-19.6 |
| **Male** | **51.50** | 51.1-51.9 | **51.70** | 51.3-52.1 | **50.60** | 50.2-51 | **46.40** | 46.1-46.8 | **49.70** | 49.3-50.1 | **46.30** | 46-46.6 | **47.00** | 46.7-47.4 | **44.90** | 44.6-45.2 | **42.80** | 42.5-43.1 | **39.20** | | 39.1-39.7 | **36.10** | | 35.8-36.3 | **45.50** | | 45.4-45.6 |
| **Age group (years)** |  |  |  |  |  |  |  |  |  |  |  |  |  |  |  |  |  |  |  | |  |  | |  |  | |  |
| **<55** | **0.8** | 0.8-0.9 | **0.70** | 0.7-0.8 | **0.80** | 0.7-0.8 | **0.70** | 0.7-0.8 | **0.80** | 0.7-0.8 | **0.70** | 0.7-0.8 | **0.70** | 0.7-0.8 | **0.80** | 0.7-0.8 | **0.70** | 0.7-0.8 | **0.70** | | 0.7-0.6 | **0.60** | | 0.6-0.7 | **0.60** | | 0.6-0.7 |
| **55-59** | **11.4** | 11-11.8 | **10.50** | 10.2-10.9 | **11.90** | 11.5-12.3 | **10.40** | 10.1-10.8 | **11.60** | 11.2-12 | **11.30** | 10.9-11.6 | **11.20** | 10.9-11.6 | **11.60** | 11.2-12 | **10.90** | 10.5-11.3 | **9.40** | | 9.1-9.4 | **9.20** | | 8.9-9.5 | **9.20** | | 8.9-9.5 |
| **60-64** | **22.8** | 22.2-23.4 | **21.70** | 21.2-22.3 | **23.60** | 23-24.2 | **21.70** | 21.2-22.3 | **25.30** | 24.7-25.9 | **24.50** | 23.9-25.1 | **25.30** | 24.7-25.8 | **26.00** | 25.4-26.6 | **24.80** | 24.3-25.4 | **22.50** | | 22.0-22.6 | **21.40** | | 20.9-21.9 | **21.40** | | 20.9-21.9 |
| **65-69** | **41.8** | 40.9-42.7 | **42.10** | 41.2-43 | **43.10** | 42.2-43.9 | **40.10** | 39.3-40.9 | **45.10** | 44.2-46 | **43.50** | 42.7-44.4 | **45.80** | 44.9-46.6 | **45.70** | 44.9-46.6 | **45.80** | 44.9-46.6 | **43.00** | | 43.5-42.7 | **41.70** | | 40.9-42.5 | **41.70** | | 40.9-42.5 |
| **70-74** | **71.1** | 69.9-72.3 | **71.00** | 69.8-72.2 | **75.40** | 74.1-76.6 | **69.40** | 68.2-70.6 | **78.20** | 76.9-79.4 | **76.80** | 75.6-78.1 | **79.20** | 77.9-80.4 | **79.00** | 77.8-80.3 | **77.00** | 75.8-78.2 | **70.00** | | 71.4-70.2 | **68.10** | | 67-69.2 | **68.10** | | 67-69.2 |
| **75-79** | **132.7** | 130.8-134.7 | **133.50** | 131.6-135.4 | **131.90** | 130.1-133.8 | **120.40** | 118.7-122.2 | **130.30** | 128.5-132.1 | **122.80** | 121.1-124.5 | **127.20** | 125.4-128.9 | **126.50** | 124.8-128.3 | **123.10** | 121.4-124.8 | **108.10** | | 112-110.4 | **110.10** | | 108.4-111.7 | **110.10** | | 108.4-111.7 |
| **80-84** | **242.4** | 239.2-245.5 | **251.70** | 248.6-254.9 | **238.30** | 235.3-241.3 | **220.20** | 217.3-223 | **234.70** | 231.9-237.6 | **215.00** | 212.3-217.8 | **219.70** | 217-222.4 | **207.10** | 204.5-209.7 | **197.20** | 194.7-199.7 | **167.30** | | 179.8-177.4 | **163.60** | | 161.4-165.9 | **163.60** | | 161.4-165.9 |
| **> 85** | **493** | 487.6-498.5 | **506.40** | 501-511.7 | **492.00** | 486.8-497.2 | **451.80** | 447.1-456.5 | **488.20** | 483.4-492.9 | **451.30** | 446.8-455.7 | **470.90** | 466.5-475.4 | **439.20** | 435-443.4 | **418.70** | 414.7-422.8 | **331.90** | | 382.1-378.3 | **339.70** | | 336.2-343.3 | **339.70** | | 336.2-343.3 |

Table S5. Average annual percentage change in standardised mortality rates for COPD in Europe with and without UK data stratified by sex and age over different time periods. Average annual percentage change data are represented as percentages. CI: confidence interval; AAPC: average annual percentage change; APC: annual percentage change; p<0.05*

|  | **AAPC 2011-2021 with UK (%)** | **95% CI** | **APC 2011-2018 with UK (%)** | **95% CI** | **APC 2019-2021 with UK (%)** | **95% CI** | **AAPC 2011-2021 without UK (%)** | **95% CI** | **APC 2011-2018 without UK (%)** | **95% CI** | **APC 2019-2021 without UK (%)** | **95% CI** |
| --- | --- | --- | --- | --- | --- | --- | --- | --- | --- | --- | --- | --- |
| **Total population** | -2.9* | -4.4-(-1.6) | -0.7 | -1.8-0.8 | -9.9* | -16.3-(-6.4) | -1.7* | -2.7-(-0.8) | -0.3 | -1.3-1.8 | -6.4* | -11.7-(-3.5) |
| **Sex** |  |  |  |  |  |  |  |  |  |  |  |  |
| Female | -2.2* | -3.7-(-0.8) | 1.1* | 0.1-2.5 | -10.0* | 13.4-(-7.2) | -0.3 | -1.4-0.7 | 1.8* | 0.9-3.2 | -6.0* | -10.7-(-3.0) |
| Male | -3.8* | -5.2-(2.5) | -2.0* | -2.6-(-1.1) | -9.0* | -13.2-(-7.3) | -3.0* | -3.5-(-2.5) | -1.9* | -2.6-(-0.1) | -7.1* | -11.4-(-4.7) |
| **Age (years)** |  |  |  |  |  |  |  |  |  |  |  |  |
| <55 | -3.0* | -5.6-(-0.5) | 0.6 | -0.6-4.7 | -10.0* | -18.8-(-3.5) | -1.84* | -3.3-(-0.3) | -0.2 | -3.4-3.0 | -5.8* | -17.9-(-8.0) |
| 55-59 | -2.5* | -4.3-(-0.8) | 0.4 | -1.0-2.8 | -10.2* | -17.3-(-5.6) | -1.70* | -3.0-(-0.4) | 0.4 | -1.0-4.7 | -8.2* | -15.9-(-3.4) |
| 60-64 | -1.3 | -2.6-0.01 | 1.7* | 0.5-3.4 | -9.0* | -14.9-(-5.2) | 0.1 | -1.1-1.2 | 2.5* | 1.1-5.0 | -6.5* | -13.3-(-2.4) |
| 65-69 | -1.1 | -2.5-0.4 | 1.1* | 0.2-2.3 | -6.6* | -11.4-(-3.9) | 0.6 | -0.2-1.5 | 1.3* | 0.6-4.5 | -5.4* | -9.2-(-0.4) |
| 70-74 | -1.7 | -3.5-0.05 | 2.4* | 1.0-4.4 | -7.3* | -10.7-(-4.8) | 0.0 | -0.7-0.7 | 1.8* | 0.7-3.6 | -5.8* | -11.5-(-2.3) |
| 75-79 | -2.8* | -4.4-(-1.3) | -0.6 | -1.7-1.0 | -8.7* | -14.7-(-5.3) | -1.3* | -2.0-(-0.6) | -0.8 | -1.9-3.5 | -5.6* | -10.8-(2.5) |
| 80-84 | -4.7* | -6.4-(-3.2) | -2.4 | -3.6-0.4 | -11.0* | -20.1-(-6.6) | -3.5* | -4.7-(-2.2) | -2.4 | -3.9-7.6 | -9.5* | -19.1-(-4.7) |
| > 85 | -4.1* | -5.2-(-3.1) | -2.1* | -3.1-(-1.1) | -10.1* | -14.6-(-7.1) | -3.0* | -4.0-(-2.1) | -1.7 | -3.3-3.2 | -11.5* | -20.0-(6.1) |

Table S6. Prevalence of Tobacco consumption across 27 European countries from 2011-2019. Data are represented as percentages of total population

| **Country** | **Smoking prevalence %** | **95% CI** |
| --- | --- | --- |
| Austria | **32** | 31-33 |
| Belgium | **24** | 23-25 |
| Bulgaria | **39** | 37-40 |
| Croatia | **36** | 35-37 |
| Czech Republic | **29** | 29-30 |
| Denmark | **23** | 22-24 |
| Estonia | **28** | 27-29 |
| Finland | **22** | 21-23 |
| France | **34** | 33-35 |
| Germany | **27** | 26-28 |
| Greece | **40** | 39-41 |
| Hungary | **32** | 31-34 |
| Ireland | **24** | 23-25 |
| Italy | **24** | 23-25 |
| Latvia | **34** | 33-35 |
| Lithuania | **29** | 28-30 |
| Luxembourg | **25** | 23-26 |
| Netherlands | **24** | 23-24 |
| Norway | **19** | 18-20 |
| Poland | **29** | 28-30 |
| Portugal | **28** | 27-29 |
| Slovakia | **27** | 26-28 |
| Slovenia | **28** | 27-30 |
| Spain | **31** | 30-32 |
| Sweden | **14** | 13-14 |
| Switzerland | **27** | 25-28 |
| United Kingdom | **23** | 22-23 |
| All Europe | **28** | 27-29 |

Table S7. Percentage change in age standardised tobacco consumption prevalence from 1990-2019 stratified by countries in Europe. CI: confidence interval

| **Country** | **% change in age standardised smoking prevalence** | **95% CI** |
| --- | --- | --- |
| Austria | **-12** | -17-(-6) |
| Belgium | **-35** | -38-(-31) |
| Bulgaria | **-18** | -25-(-12) |
| Croatia | **-15** | -21-(-8) |
| Czechia | **-21** | -26-(-15) |
| Denmark | **-49** | -52-(-46) |
| Estonia | **-22** | -26-(-17) |
| Finland | **-29** | -34-(-24) |
| France | **-10** | -14-(-6) |
| Germany | **-19** | -23-(-15) |
| Greece | **-11** | -15-(-7) |
| Hungary | **-20** | -26-(-14) |
| Ireland | **-34** | -38-(-29) |
| Italy | **-29** | -33-(-25) |
| Latvia | **-9** | -15-(-2) |
| Lithuania | **-5** | -12-1 |
| Luxembourg | **-30** | -35-(-25) |
| Netherlands | **-44** | -47-(-41) |
| Norway | **-53** | -58-(-49) |
| Poland | **-32** | -35-(-28) |
| Portugal | **-3** | -9-2 |
| Slovakia | **-20** | -27-(-12) |
| Slovenia | **-3** | -12-7 |
| Spain | **-33** | -36-(-29) |
| Sweden | **-46** | -51-(-40) |
| Switzerland | **-21** | -26-(-16) |
| United Kingdom | **-39** | -42-(-35) |
| All Europe | **-25** | -29-(-19) |


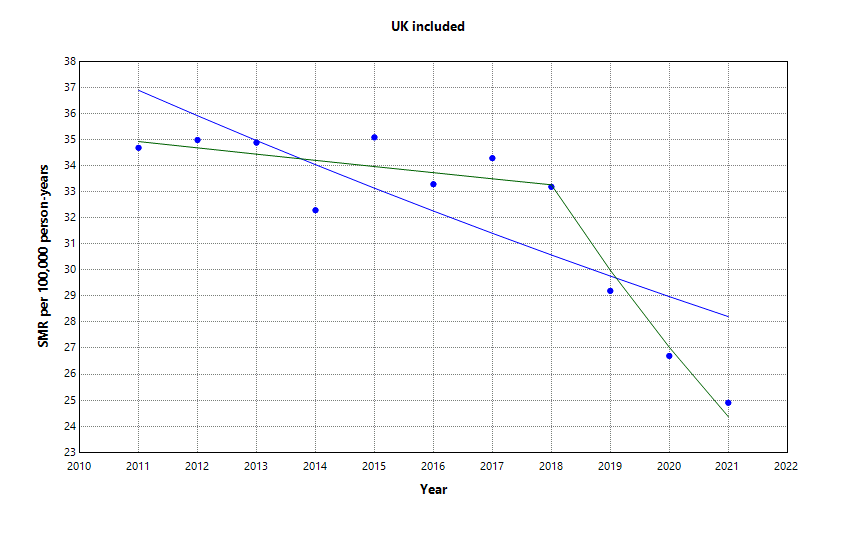
a)


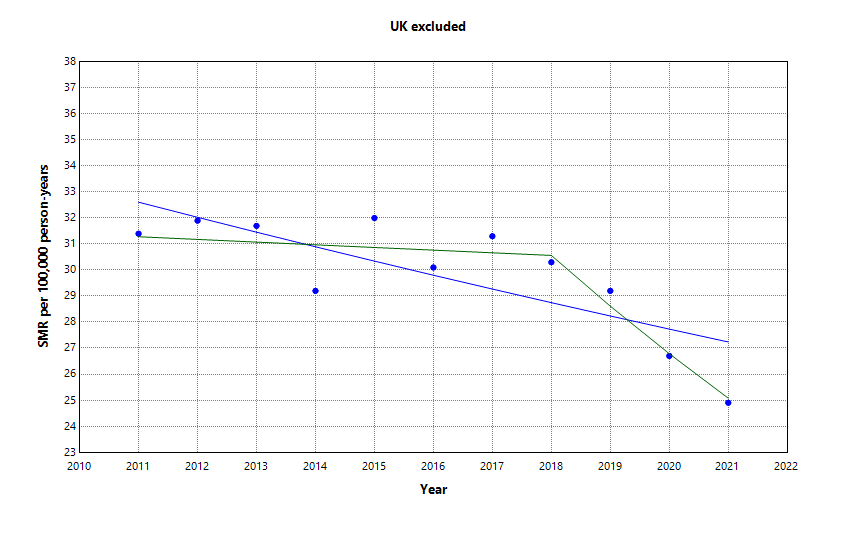
b)

Figure S1. Joinpoint regression graphs for age- and sex- standardised mortality rates for COPD in Europe from 2011-2021 a) with and b) without UK data. The blue line represents the trend from segmented regression model with 0 joinpoints, the green line represents the trend from the segmented regression with 1 joinpoint and the symbols are the raw data expressed per 100,000 person-years. SMR: standardised mortality rates.

Figure S2. Joinpoint regression graphs for standardised mortality rates for COPD in 12 European countries (Belgium, Germany, United Kingdom, France, Italy, Greece, Spain, Poland, Netherlands, Sweden, Czech Republic, and Portugal) with a population of more than 10million people from 2013-2019. The blue line represents the trend from segmented regression model with 0 joinpoints and the symbols are the standardised mortality rate data expressed per 100,000 person-years.

Figure S3. Age- and sex- standardised COPD related annual mortality rates from 2011-2021 for 27 European countries
